# A Rare Case of Severe Mitral Stenosis Presenting As Cardiogenic Shock

**DOI:** 10.7759/cureus.57627

**Published:** 2024-04-04

**Authors:** Lucas Garcia Reinoso, Sabu John

**Affiliations:** 1 Internal Medicine, State University of New York Downstate Health Sciences University, Brooklyn, USA; 2 Cardiology, State University of New York Downstate Medical Center, Brooklyn, USA

**Keywords:** pocus (point-of-care ultrasound), preoperative cardiac evaluation, rheumatic mitral stenosis, severe mitral stenosis, cardiology inpatient/outpatient consult

## Abstract

We report a case of severe mitral stenosis (MS) in a 58-year-old female from Guyana. Though rheumatic MS continues to be less prevalent in third-world countries, it poses a significant threat as far as morbidity and mortality are concerned. The modern definition of “Third World” is used to classify countries that are poor or developing. Countries that are part of the “third world” are generally characterized by (1) high rates of poverty, (2) economic and/or political instability, and (3) high mortality. The standard method of diagnosing MS in patients has been established as transthoracic echocardiograms (TTE), along with pertinent historical and physical exam findings. Specifically, with TTE, criteria include a mitral valve area ≤1.5 cm^2^, severe left atrial enlargement, and elevated pulmonary artery systolic pressure >50 millimeters of mercury (mmHg). Once diagnosed with severe MS, treatment options for patients range from non-surgical percutaneous mitral balloon valvuloplasty to surgical mitral valve commissurotomy. In our case, she was a 58-year-old female with a past medical history of seizures of unknown etiology, not on any home medication regimen, presenting to the emergency department with shortness of breath, malaise, weight loss, and bilateral lower leg edema. Vitals were significant for tachycardia at 153 bpm, tachypnea at 24 breaths per minute, and saturating at 96% on room air. On the physical exam, there was an irregularly irregular rhythm, bilateral crackles at the bases, right upper quadrant tenderness to palpation, bilateral pitting edema, and no calf tenderness. Lab findings were significant for elevated brain natriuretic peptide, but three electrocardiograms were performed in the emergency department, all confirming the new onset of atrial fibrillation. A CT angiogram of the chest was performed, which ruled out pulmonary embolus but additionally found marked reflux of contrast noted within the inferior vena cava and hepatic veins, along with right atrial dilation reflective of right heart strain; additionally, mitral valve calcifications were noted. The cardiologist on duty confirmed the diagnosis using point-of-care ultrasound (POCUS) followed by TTE; the patient was rapidly transferred to a hospital with appropriate services for surgical management within the same day of arrival at the emergency department. This case highlights the importance of bedside POCUS as an additional diagnostic tool for cardiologists, along with pertinent history, physical examination findings, and laboratory findings. Proper utilization of POCUS can allow for the immediate diagnosis of severe pathologies and prevent the delay of appropriate treatment, as seen in our case. Wider adoption of POCUS practices as a part of the general initial evaluation of patients has not yet been recommended by the American Heart Association but can offer clinical benefit in morbidity/mortality with expedited progression to appropriate treatment.

## Introduction

Mitral stenosis (MS) secondary to rheumatic heart disease, though decreasing in prevalence, continues to be a significant cause of morbidity and mortality in developing countries worldwide. Recent studies have found trends of incidence and prevalence in low and middle socio-demographic index (SDI) regions and countries have been increasing, 0.30, when compared to decreasing trends in high-middle and high SDI -0.58 [1]. Nonrheumatic MS with mitral annular calcification has been increasing in recent years owing to the aging population as well, increasing the importance of an appropriate diagnosis. Untreated, severe MS carries morbidity and mortality with limited therapeutic options; in the asymptomatic or minimally symptomatic patient, survival is greater than 80% at 10 years, yet when limiting symptoms occur, 10-year survival is less than 15% in the patient with untreated MS [2]. Additional common causes of MS, which are increasing in prevalence, are survivors of mantle irradiation and advanced kidney disease [3].

Rheumatic MS demographically is more common in women than men with around ~80% prevalence of cases. Additionally, older patients, from around 50 to 70 years old, are the usual presenting age in developed countries. These patients present with calcified fibrotic leaflets along with commissural fusion and sub-valvular involvement. Being that the usual presentation is of an older age, they often have multiple comorbidities, such as atherosclerotic disease, hypertension, and diastolic dysfunction. Because of the higher comorbidity burden, evaluation for further management is complicated, and eventually, options are limited in this population [4].

Some definitions of “severe” MS are additionally based on the severity of symptoms and the severity at which interventions will improve symptoms; some examples of these presenting symptoms are decreased exercise tolerance and exertional dyspnea. A transthoracic echocardiogram (TTE) is the modality of choice to evaluate for MS. Criteria for severe MS on a TTE include a mitral valve area ≤1.5 cm^2^, severe left atrial enlargement, and elevated pulmonary artery systolic pressure >50 millimeters of mercury (mmHg) [5].

## Case presentation

The patient is a 58-year-old female with a past medical history of seizures of unknown etiology, not on any home medication regimen, presenting to the emergency department with shortness of breath, malaise, weight loss, and bilateral lower leg edema. They recently arrived from Guyana. The morning of her visit to the emergency department, it was noted that her shortness of breath had been occurring for the last year but had acutely worsened during her flight, and her leg swelling had been occurring for an unknown period of months. Additionally, the patient lost almost 50 pounds of body weight during the past year. The patient had not been to this hospital system prior to this visit to the emergency department.

Initially, the patient was tachypneic and tachycardic to 24 and 153 bpm, respectively, while saturating at 96% on room air with increased work of breathing. He was recently detected hypertensive with a BP of 133/105 mmHg but afebrile. On physical examination, pertinent findings were irregularly irregular rhythm, bilateral crackles at the bases, right upper quadrant tenderness to palpation, bilateral pitting edema, and no calf tenderness; she was alert and oriented to person, place, time, and situation. The emergency department team performed a point-of-care ultrasound (POCUS) examination, which resulted in three or more B lines in each lung window, right-sided pleural effusion, and small pericardial effusion with questionable evidence of right-sided heart strain, in their opinion. This caused them to proceed to CT angiogram for pulmonary embolism (PE) to rule out pulmonary embolus. Three electrocardiograms were performed in the emergency department, which confirmed the first recorded instance of atrial fibrillation. A CT angiogram PE was performed and was negative for pulmonary embolus. Additionally, a right lower lobe consolidative opacity was questionable for pneumonia versus pulmonary infarct, left lower lobe and right upper lobe peripheral opacities were questionable evidence for atelectasis versus multifocal pneumonia, a right-sided pleural effusion was found, and lastly, there was marked reflux of contrast noted within the inferior vena cava and hepatic veins along with right atrial dilation reflective of right heart strain; the second CTs performed confirmed the previous examination results along with additional mitral valve calcifications noted. CT abdomen/pelvis with intravenous (IV) contrast performed exhibited evidence for passive hepatic congestion secondary to right heart dysfunction, with gastric variation in the setting of portal hypertension and a hypoattenuating partially calcified right renal lesion suspicious for neoplasm.

**Figure 1:**
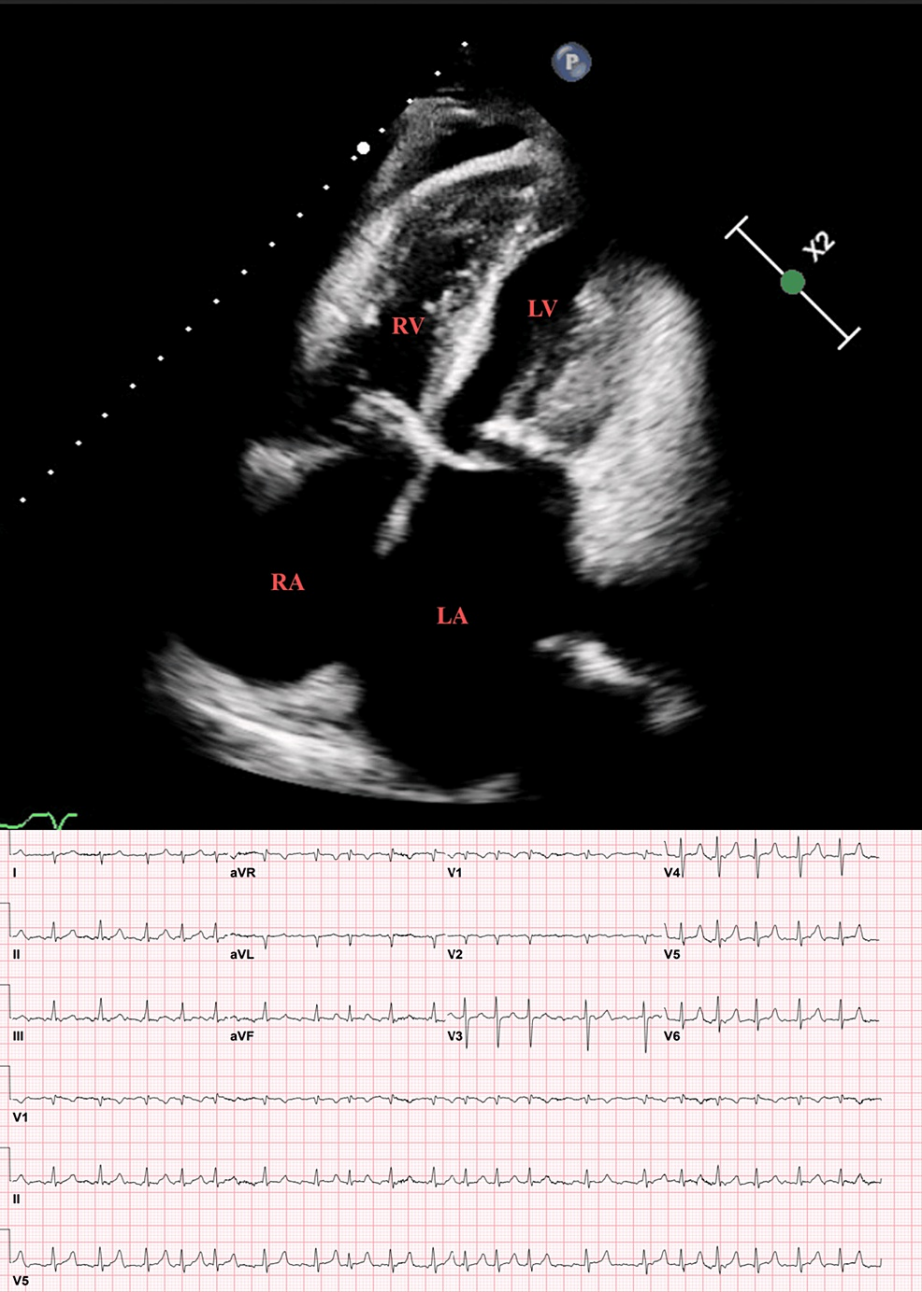
Four-chamber apical view exhibiting thickened mitral valve leaflets and marked dilation of left atrium. The electrocardiogram at the time of ultrasound displayed atrial fibrillation LA: left atrium, LV: left ventricle, RA: right atrium, RV: right ventricle

**Figure 2:**
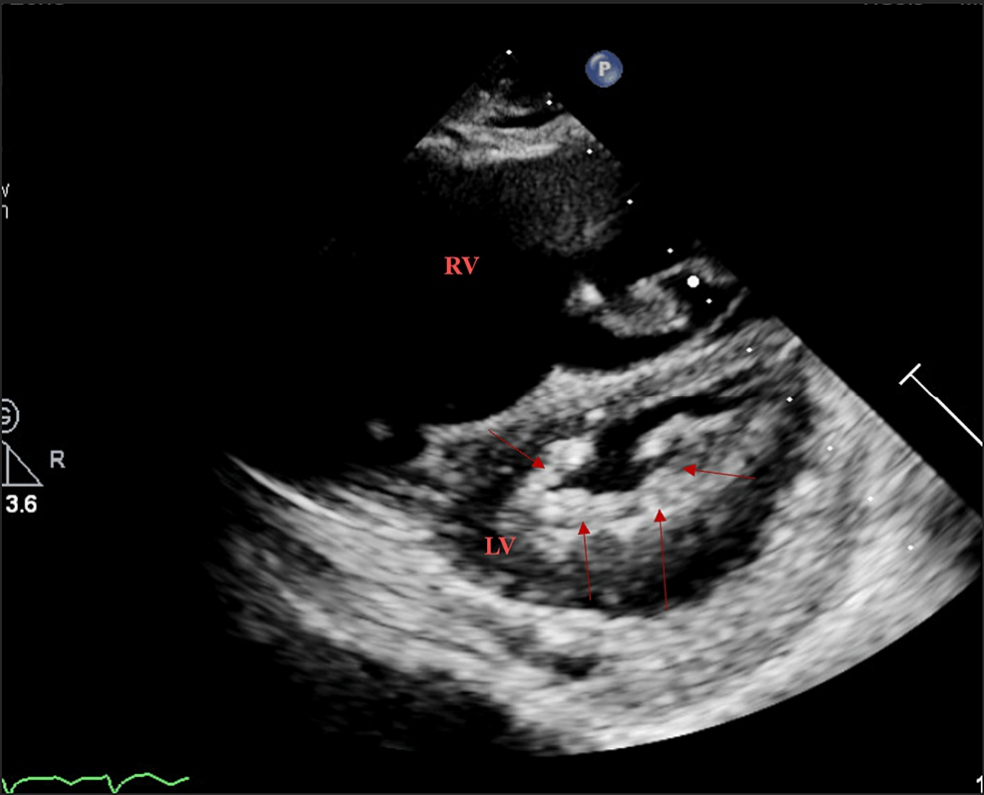
Parasternal short-axis with restricted mitral orifice and diffuse thickening surrounding the orifice (arrows) RV: right ventricle, LV: left ventricle

**Figure 3:**
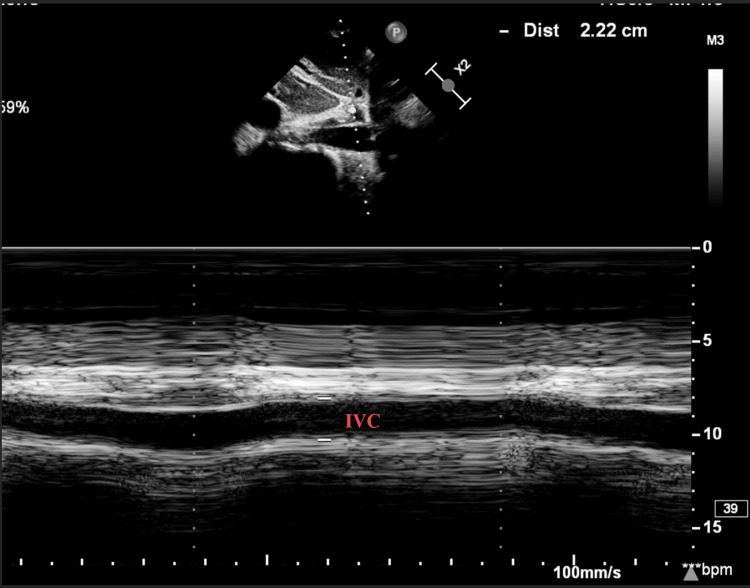
Inferior vena cava dilated to 2.2 cm IVC: inferior vena cava

**Figure 4:**
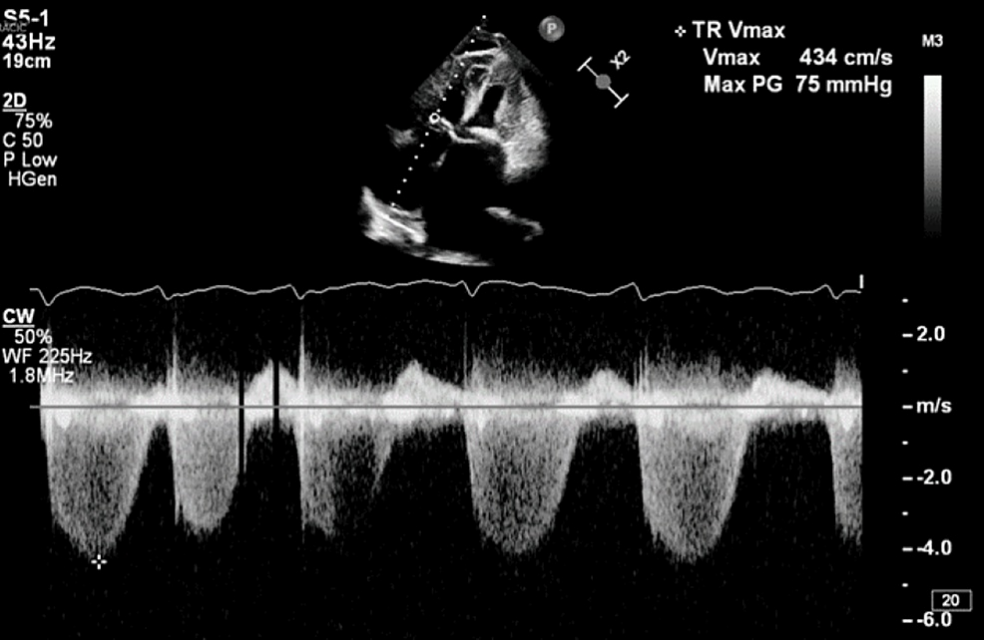
Elevated TR velocity consistent with pulmonary hypertension. The TR velocity is 4.34 m/s. IVC is dilated without respiratory variation suggesting elevated RA pressure to 15 mmHg calculated PASP is 90 mmHg TR: tricuspid regurgitation, IVC: inferior vena cava, RA: right atrial, PASP: pulmonary arterial systolic pressure

**Figure 5:**
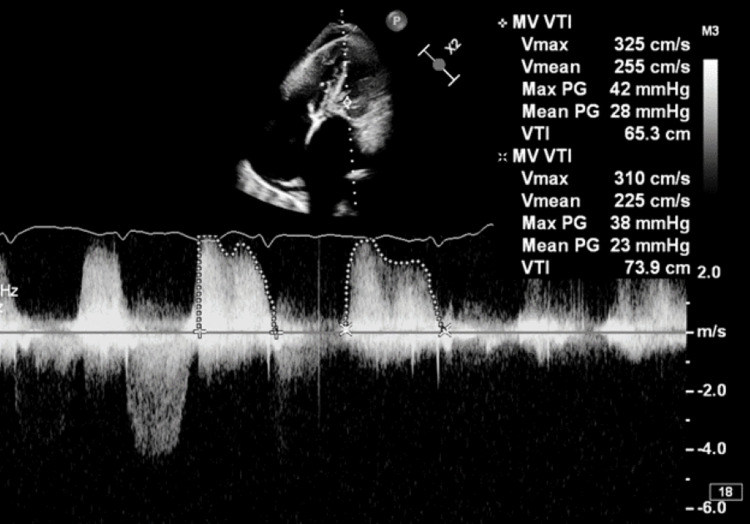
Mean mitral gradient of 28 mmHg is consistent with severe mitral stenosis

While in the emergency room, the patient was found to have an episode of altered mental status and questionable respiratory distress with decreasing oxygen saturation; upon review of her telemetry monitoring, she was found to be in atrial fibrillation with a rapid ventricular rhythm ranging from 80 to 170 bpm. The cardiology service was then consulted and performed a bedside POCUS, which was highly suspicious for severe MS (Figures 1-5). POCUS results guided recommendations for the patient to be transferred to full-dose anticoagulation of Coumadin with a heparin bridge; TTE performed indicated possible initiation of amiodarone for rate control; aggressive diuresis with right heart catheterization for hemodynamic evaluation and diuresis assistance; and urgent transfer to a different hospital for possible mitral valve commissurotomy/replacement versus percutaneous mitral balloon valvuloplasty (PMBV) due to suspected rheumatic MS.

The patient then proceeded to the hospital transfer on the same day. There, the patient underwent placement of the right arterial line and Swan-Ganz catheter by the cardiac critical care unit (CCU) team. Esmolol and nitroprusside drips were administered due to a continued heart rate of 150-180 beats per minute and mean arterial pressures around 100 millimeters of mercury (mmHg). Additionally, 120 milliliters (ml) of Lasix (furosemide) IV were administered, calculated at a dose of two milliliters per kilogram (kg) as she weighed 60 kg. The patient then became agitated and confused, unable to tolerate bilevel positive airway pressure. After a discussion with the family, who requested a full-code designation, they were intubated. Immediately after intubation, the patient had a cardiac arrest (pulseless electrical rhythm), which led to five cycles of cardiopulmonary resuscitation performed with the resuscitation of spontaneous circulation achieved after 10 minutes, during which they did not have shockable rhythms.

A transesophageal echocardiogram was then performed on the patient, which was technically difficult but exhibited severe left atrial dilation with left atrial appendage thrombus, severe right atrial dilation, an underfilled/small left ventricular cavity with difficulty assessing function due to tachycardia, a non-significantly dilated right ventricle with similar difficulties assessing function, severe mitral valve thickening with calcification and a mean gradient >20 mmHg, moderate tricuspid regurgitation, a small pericardial effusion, and a pulmonary artery systolic pressure of 90 mmHg categorized as severe pulmonary arterial hypertension. Heparin was begun for the patient at 12 units per kg per hour, as well as digoxin, while phenylephrine at 25 micrograms (mcg) per minute (min) + norepinephrine at 4 mcg/min were continued to maintain mean arterial pressure ~100 mmHg.

Cardiothoracic surgery was consulted, which recommended possible PMBV with possible veno-arterial extracorporeal membrane oxygenation (ECMO) placement. The patient was placed on ECMO and hemodynamically improved, which deferred PMBV for bridge-to-valve surgery. Left-heart catheterization was performed, resulting in mild, non-obstructive coronary artery disease. The video electroencephalogram performed showed questionable epileptiform discharges, and the CCU team proceeded to the CT head, which displayed an 11-millimeter hemorrhagic stroke at the right precentral gyrus (previous history of seizures may be related to cavernous malformation hemorrhagic lesion); after discussion with cardiothoracic surgery, the patient was determined to be a poor candidate for surgical intervention. Returning to the PMBV intervention, cardiothoracic surgery placed an Impella ventricular pump for expected worsened mitral regurgitation after valvuloplasty. The patient then failed the PMBV procedure with continued severe MS and returned from the operating room to the CCU in multi-pressor shock. It was determined that the patient should proceed to an intra-aortic balloon pump and be weaned off ECMO as they were no longer a candidate. The procedure was successful, and neurology/cardiothoracic surgery was planned for possible surgical MS replacement.

## Discussion

PMBV is a non-surgical procedure that introduces a balloon to the mitral valve orifice and is inflated to fracture the calcified commissures of the valve, increasing the valvular area. In comparison to surgical mitral commissurotomy, PMBV has shown equal or better success rates and comparable restenosis rates. The most widely used echocardiographic parameter to determine eligibility for PMBV is the Wilkins score, which reviews the anatomy of the leaflet, the commissures, and the sub-valvular apparatus. The scoring system assigns a point value from 1 to 4 for each of (1) valve calcification, (2) leaflet mobility, (3) leaflet thickening, and (4) sub-valvular apparatus degeneration. A mitral valve with a score <8 to 9 and no more than moderate mitral regurgitation is deemed the best candidate for PMBV. In patients with a score >9 to 10, especially with more than moderate mitral regurgitation, surgical therapy should be advised except in cases with serious comorbidities, comparable to our patient who scored a 10 yet had serious comorbidities. PMBV theoretically functions by splitting the commissures open from calcification, increasing the valve area, and allowing for increased flow [5]. A valve with a bilateral commissural fusion from calcification would respond better, whereas a valve where rigid leaflets or annulus give rise to orifice stenosis may not respond to PMBV; the leaflets or sub-valvular apparatus could fracture due to the rigidity, causing emergent adverse effects requiring immediate valve replacement. The degree and amount of calcification of the commissures, such as severe or bilateral calcification on the commissures, could predict poor outcomes. This may be due to worsening mitral regurgitation post-procedurally or a suboptimal increase in the valve area [6].

A major contraindication of PMBV is thrombus presence in the left atrial appendage due to the danger of embolization caused by the manipulation of guidewires and balloon catheters. Ideally, PMBV should be postponed when a thrombus is discovered, and the patient should be started on warfarin with the prothrombin time-international normalized ratio at a therapeutic dose for three to six months. TTE should be repeated to confirm the disappearance of the clot [7,8]. If the thrombus has resolved, PMBV can then be safely performed; if the thrombus remains, the recommendation is surgical intervention on the mitral valve along with removal of the thrombus. Other contraindications to PMBV are more than moderate mitral regurgitation and massive or bi-commissural calcification, as previously discussed; severe concomitant aortic valve disease; severe organic tricuspid stenosis or severe functional regurgitation with an enlarged annulus; or severe concomitant coronary artery disease requiring bypass surgery [9].

Mitral valve surgery therapy for rheumatic MS is another option, with the preferred approach being commissurotomy, either closed, where the valve is opened blindly through the left atrium or left ventricle, or open, usually reserved for when more extensive repair is necessary and there is optimal anatomy. "Closed mitral valvotomy, though historically significant, provided satisfactory results" [10]. When there is severe valvular thickening and sub-valvular fibrosis with leaflet tethering, mitral valve replacement may be the best option. In addition to those who have suboptimal valve anatomy or have failed PMBV, patients with moderate or severe tricuspid regurgitation can also have better outcomes with surgical intervention, as tricuspid valve repairs can be performed simultaneously. Because rheumatic MS slows progression over decades, surgery should be delayed until the patient has severe limiting symptoms, classified as meeting New York Heart Association class III or IV criteria, particularly if mitral valve repair is proposed. Some patients will have developed intrinsic pulmonary vascular disease at later stages, which are poor outcome predictors, as evidenced when comparing long-term survival rates. Contraindications of this procedure are when patients are unable to tolerate systemic anticoagulation or if there is an underlying bleeding disorder with coagulopathy [9,11].

Even though both of these procedures, when successful, significantly improve morbidity and mortality outcomes for patients, it is imperative that both catheter-based and surgical procedures are performed at experienced centers in order to limit intra-operative complications. For instance, in the United States, there has been a 7.5% decrease in the use of PMBC, accompanied by a 15.9% increase in the complication rate [12]. Additionally, excellent short- and long-term outcomes can be achieved with surgical mitral valve repair, but the procedure is not routinely or widely performed by most surgeons in the United States. Thus, in the clinical decision-making process for a patient with rheumatic MS, it is essential to know the results of the available interventional procedures to determine appropriate risk.

Due to increasing access and technological advances, POCUS has become an essential tool for physicians to use while approaching clinical decision-making. Core training has begun to incorporate POCUS curriculum due to the additional information that can be used in decision-making, possibly decreasing the time between diagnosis and life-sustaining treatment options in the acute setting. As the worldwide population age continues to increase, valvular heart disease incidence, morbidity, and mortality will expectedly increase as well [13]. Some reviews, such as Thomas et al. al., found evidence that medical students with brief training could recognize rheumatic mitral valve abnormalities with a sensitivity of 81% and a negative predictive value of 89% [14]. Even when compared to traditional auscultation by stethoscope, handheld ultrasound devices performing POCUS TTEs were able to detect early signs of valvular heart disease in 100% of cases with experienced physicians [15]. Studies such as Wen and Naqvi were able to compile data showing that the degree of severity for valvular heart disease was significantly better with pocket ultrasound machines when compared to auscultation; though limited due to sample size, POCUS was able to diagnose severity and lead to appropriate repair procedures following the official TTE diagnosis [16].

Though POCUS has been found to be a generally positive diagnostic tool for physicians, there are some limitations that can limit its efficacy for application. General limitations occur initially with training and mastery of the POCUS technique and recognition of pathological findings. Additionally, the types of POCUS machines available at any hospital can limit effectiveness due to access to functionality, image clarity, and maintenance. Lastly, human error can cause misdiagnosis or confusion, which can delay treatment, as seen in the study by Doufle et al., where misdiagnosed POCUS readings led to delayed treatment of life-threatening cardiac tamponade following a surgical procedure [17].

## Conclusions

This case highlights the importance of bedside POCUS as an additional tool for cardiologists to diagnose patients along with pertinent history, physical examination findings, and laboratory findings. Proper utilization of POCUS can allow for the immediate diagnosis of severe pathologies and prevent the delay of appropriate treatment. Wider adoption of POCUS practices as a part of the general initial evaluation of patients has not yet been recommended by the American Heart Association but offers clinical benefit in morbidity/mortality with expedited progression to appropriate treatment. Though an important tool for diagnosis, careful consideration in regards to training, equipment, and human error must be accounted for when being implemented; official TTE readings will always be required for confirmatory studies and the determination of further treatment plans.

Our patient presented a uniquely difficult situation where their presumed rheumatic mitral regurgitation initially presented with a possible PMBC candidate but was complicated by a transfer process that delayed proper treatment. Additionally, upon further review of echocardiogram results, the patient carried many contraindications to percutaneous mitral balloon commissurotomy due to severe calcification of the annulus and commissures, severely elevated pulmonary artery pressures, standing moderate mitral regurgitation, and a left atrial appendage thrombus, all during an emergently deteriorating clinical course. Furthermore, when mitral valve surgical commissurotomy/replacement was reconsidered, the patient unfortunately sustained a hemorrhagic stroke, which delayed surgical intervention further until anticoagulation was approved by the neurology team after multidisciplinary discussions. The occurrence of the symptomatic and inpatient contraindications during the hospitalization led to the mitral valve repair being delayed until PMBV failure. Once stabilized, the cardiothoracic team decided on mitral valve replacement, and the patient has been able to be discharged, recovering with great neurologic recovery and cardiac function since.

Due to the poor post-surgical prognosis and significant adverse events that have occurred, retrospective reflection warrants a review of whether delayed surgical intervention, in view of high pre-operational risks, ultimately negatively impacted outcomes in severely emergent cases such as this. The expectation is that surgical mitral valve repair will successfully prevent further heart failure and allow for optimal neurologic recovery.
